# Striatal dopamine synthesis capacity in autism spectrum disorder and its relation with social defeat: an [^18^F]-FDOPA PET/CT study

**DOI:** 10.1038/s41398-020-01174-w

**Published:** 2021-01-13

**Authors:** Rik Schalbroeck, Floris H. P. van Velden, Lioe-Fee de Geus-Oei, Maqsood Yaqub, Therese van Amelsvoort, Jan Booij, Jean-Paul Selten

**Affiliations:** 1 Rivierduinen Institute for Mental Healthcare, Leiden, The Netherlands; 2 School for Mental Health and Neuroscience, Maastricht University, Maastricht, The Netherlands; 3 Section of Nuclear Medicine, Department of Radiology, Leiden University Medical Center, Leiden, The Netherlands; 4 Biomedical Imaging Group, University of Twente, Enschede, The Netherlands; 5 Department of Radiology and Nuclear Medicine, Amsterdam University Medical Centers, location Vrije Universiteit Medical Center, Amsterdam, The Netherlands; 6 Department of Radiology and Nuclear Medicine, Amsterdam University Medical Centers, location Academic Medical Center, Amsterdam, The Netherlands

**Keywords:** Schizophrenia, Neuroscience, Autism spectrum disorders

## Abstract

Alterations in dopamine signalling have been implied in autism spectrum disorder (ASD), and these could be associated with the risk of developing a psychotic disorder in ASD adults. Negative social experiences and feelings of social defeat might result in an increase in dopamine functioning. However, few studies examined dopamine functioning in vivo in ASD. Here we examine whether striatal dopamine synthesis capacity is increased in ASD and associated with social defeat. Forty-four unmedicated, non-psychotic adults diagnosed with ASD and 22 matched controls, aged 18–30 years, completed a dynamic 3,4-dihydroxy-6-[^18^F]-fluoro-L-phenylalanine positron emission tomography/computed tomography ([^18^F]-FDOPA PET/CT) scan to measure presynaptic dopamine synthesis capacity in the striatum. We considered unwanted loneliness, ascertained using the UCLA Loneliness Scale, as primary measure of social defeat. We found no statistically significant difference in striatal dopamine synthesis capacity between ASD and controls (*F*_1,60_ = 0.026, *p* = 0.87). In ASD, striatal dopamine synthesis capacity was not significantly associated with loneliness (*β* = 0.01, *p* = 0.96). Secondary analyses showed comparable results when examining the associative, limbic, and sensorimotor sub-regions of the striatum (all *p*-values > 0.05). Results were similar before and after adjusting for age, sex, smoking-status, and PET/CT-scanner-type. In conclusion, in unmedicated, non-psychotic adults with ASD, striatal dopamine synthesis capacity is not increased and not associated with social defeat.

## Introduction

Adults with autism spectrum disorder (ASD) are at an increased risk of developing a psychotic disorder^1^. However, risk factors and neurobiological alterations associated with this risk remain poorly understood^2^.

A well-replicated finding of neurotransmitter functioning in psychosis has been that of increased presynaptic dopamine functioning. In vivo molecular imaging studies using positron emission tomography (PET) or single-photon emission computed tomography (SPECT) have consistently shown that psychosis is strongly related to increased presynaptic dopamine synthesis capacity and endogenous dopamine release in the striatum^3^. This increase in presynaptic dopamine functioning has also been reported in individuals at-risk of psychotic disorder^4,5^ and in individuals with a non-psychotic disorder when psychotic symptoms are present^6^.

Several researchers have suggested that alterations in dopamine functioning exist in ASD^7^. For instance, this has been implied in theoretical work^8^, gene studies^9^, and studies examining dopamine-modulating medication^10,11^. However, molecular imaging studies, in particular of presynaptic dopamine, are lacking^12^. No studies examined striatal dopamine release, and only two studies used 3,4-dihydroxy-6-[^18^F]-fluoro-L-phenylalanine ([^18^F]-FDOPA) PET to measure dopamine synthesis capacity. The first^13^ found no significant increase in striatal dopamine synthesis capacity in autistic children (*n* = 14) relative to non-autistic peers (*n* = 10), but most (*n* = 11) autistic children were sedated using propofol which can influence dopamine concentrations^14^. The second^15^ obtained evidence of increased striatal dopamine synthesis capacity in adults with Asperger syndrome (*n* = 8) relative to controls (*n* = 5), but the number of participants was small and the influence of possible risk factors was not examined.

Evidence indicates that psychosocial stress can influence dopamine functioning^16^. According to the social defeat hypothesis of schizophrenia, the long-term subjective experience of outsider status or subordinate position leads to increased baseline activity and/or sensitization of the brain dopamine system^17–19^. In line with this hypothesis, increased presynaptic dopamine functioning has been reported in non-psychotic groups presumed to be more socially defeated, such as immigrants, individuals with severe hearing impairment, and individuals with a history of childhood trauma^20–23^. Whether social defeat also increases presynaptic dopamine functioning in individuals with ASD, who are widely exposed to defeating experiences such as loneliness^24^, bullying victimization^25,26^, and discrimination^27^, remains unknown.

Here we examine striatal presynaptic dopamine synthesis capacity, measured with [^18^F]-FDOPA PET/computed tomography (PET/CT), in non-psychotic adults with ASD and controls aged 18–30 years. We expect (hypothesis 1a) that striatal dopamine synthesis capacity is increased in ASD relative to controls. Furthermore, we expect (hypothesis 1b) that in ASD, striatal dopamine synthesis capacity is positively associated with loneliness, as an approximate measure of social defeat. As secondary outcomes, we expect (hypothesis 2a) that adults with ASD report more social defeat than controls, and (hypothesis 2b) that striatal dopamine synthesis capacity is positively related to other measures of social defeat (besides loneliness) and to subclinical psychotic symptoms. Finally, we expect (hypothesis 3) the strongest associations with dopamine synthesis capacity in the associative striatum, since the largest striatal dopaminergic disruptions in psychosis have been reported in this sub-region^3^ (with reference to hypotheses 1a-b and 2b).

## Materials and methods

### Participants

ASD and control participants were Dutch adults aged 18–30 years, recruited via online social media and poster advertisements. Adults with ASD were also recruited at mental healthcare institutes, ASD housing services, university counselling services, and ASD-related websites. All of these adults had previously been diagnosed with an ASD by a registered mental health clinician, and the first author confirmed this diagnosis using the Autism Diagnostic Observation Schedule-2 (ADOS-2) module 4^28,29^. The ASD and control samples consisted of 45 and 24 participants, respectively, which were frequency-matched on age, sex, and smoking-status (yes/no).

Exclusion criteria (explained in detail in Supplement 1) included a lifetime diagnosis of psychotic or bipolar disorder assessed by self-report, psychotic symptoms indicative of psychotic disorder assessed with the Comprehensive Assessment of At-Risk Mental States (CAARMS)^30,31^, IQ lower than 85 assessed with the Dutch Adult Reading Test^32^, or a history of alcohol- or drug abuse or dependence assessed with the Composite International Diagnostic Interview (CIDI) v2.1^33^. Additional exclusion criteria included self-reported or suspected neurological disorder, brain damage, history of meningitis, fragile X syndrome, Rett syndrome, 22q11 deletion syndrome, metal objects in or around the body, participation in a scientific examination where radiation was used in the past year, and (in females) lactation or pregnancy (confirmed with a urinary pregnancy test). Current and/or recent medication or illicit drug use, assessed through self-report, was also prohibited. A urinary test on the day of the PET/CT-scan had to be negative for opiates, cocaine, cannabis, and amphetamines.

### Sample size calculations

Sample size calculations were conducted using G*Power v3.1^34,35^, on the basis of a two-tailed alpha of 0.05 and analysis within the general linear model. The necessary sample size to examine group differences in striatal dopamine synthesis capacity was calculated using a study that compared dopamine synthesis capacity in striatal regions of adults with Asperger syndrome and controls^15^. If results from our study would have been similar to the smallest effect and the largest standard deviation (SD) of dopamine synthesis capacity values of that study (difference in means = 0.34, *SD* = 0.35), we would need between 19 and 24 participants assuming a power of 0.80–0.90. Therefore, we included 24 controls.

The necessary sample size to examine the relationship between striatal dopamine synthesis capacity and loneliness in ASD was based on a study that examined the relationship between dopamine functioning and reports of childhood trauma in healthy volunteers^21^. Assuming a similar effect size of approximately *r* = 0.4 and a power of 0.80, we would need 45 participants. Therefore, the ASD sample included 45 participants.

### Design and procedures

Participants were assessed on three separate testing days, during which they were screened for in- and exclusion criteria, underwent a magnetic resonance imaging (MRI) scan (see below), and completed measures of social defeat and an [^18^F]-FDOPA PET/CT-scan. All participants signed informed consent prior to the start of the study. The study was approved by the medical ethics committee of Leiden University Medical Center (reference NL54244.058.15), and pre-registered within the Netherlands Trial Register (registration number NL6207, https://www.trialregister.nl/trial/6207).

### Measures of social defeat and psychotic symptoms

Measures of social defeat and psychosis are described in Supplement 2. In brief, we assessed loneliness using the UCLA Loneliness Scale^36^ and used this as the primary measure of social defeat since it reflects a lack of social participation as well as the negative experience of this, in line with the definition of social defeat^19^.

As secondary measures of social defeat we assessed experiences of being ostracized using the Ostracism Experience Scale (OES)^37^, bullying victimisation (yes/no) and its total duration before age 17 using a modified version of the Olweus Bullying Interview^38^, social network size using the Lubben Social Network Scale (LSNS)^39^, and childhood trauma before age 17 using the Childhood Trauma Questionnaire (CTQ)^40,41^. Moreover, we assessed the desire for social acceptance and belonging using the Need to Belong Scale (NBS)^42^, and the perceived availability of social support using the Interpersonal Support Evaluation List (ISEL)^43^. We calculated the interaction between the NBS and ISEL and used this as a predictor variable in the analyses to examine whether a discrepancy between them was related to the outcomes.

We assessed self-reported psychotic symptoms using the Prodromal Questionnaire-16 (PQ-16)^31,44^. The PQ-16 was added to the project when data collection had already been started and was therefore completed by a subset of 48 participants (*n* = 31 ASD, *n* = 17 controls). Furthermore, we assessed depressed mood and anxiety using the Beck’s Depression Inventory-II (BDI-II)^45^ and State-Trait Anxiety Inventory-Trait subscale (STAI-T)^46^, which we used in exploratory analyses.

### MRI and PET/CT acquisition

A structural T1-weighted MRI (3D fast field echo sequence) was obtained for each participant on a 3T Ingenia MR scanner (Philips Healthcare, Best, The Netherlands). Due to a necessary replacement of the PET/CT-scanner at the hospital in which this study was conducted, PET/CT data were collected on two different PET/CT systems, i.e. a Biograph Horizon with TrueV option (Siemens Healthineers, Erlangen, Germany) or Vereos (Philips Healthcare, Best, The Netherlands). Both systems received EARL PET/CT accreditation. Reconstruction settings were harmonized using acquisitions of a Hoffman 3D brain phantom^47^ (data not shown). Participants were asked to refrain from smoking and from eating or drinking (except water) 3 and 6 h, respectively, prior to the PET/CT-scan. One hour before the scan, participants consumed 150 mg carbidopa and 400 mg entacapone to reduce the formation of radiolabelled [^18^F]-FDOPA metabolites^48,49^. Immediately prior to the PET acquisition, a low-dose CT-scan of the brain (110/120 kVp, 35 mAs) was acquired for attenuation correction purposes. Subsequently, 150 MBq [^18^F]-FDOPA was administered intravenously as a bolus, which was followed by a 90-min dynamic PET acquisition. Head movement was minimalised with a headrest and head strap, and participants were monitored throughout the PET acquisition so that any discomfort could be observed and addressed. Data were collected in list mode and histogrammed into 25 timeframes (5 × 1, 3 × 2, 3 × 3, and 14 × 5 minute(s)). PET data were reconstructed iteratively with resolution modelling and 2-mm full-width-at-half-maximum Gaussian smoothing filter with a voxel size of 2 x 2 x 2 mm^3^.

### Image processing

Image processing was conducted by researchers who were blind to ASD status. The MRI- and PET-images (except for the first two frames) were rigidly co-registered to a single PET-frame acquired 7 min post-injection using Vinci (v4.83; Max Planck Institute for Neurological Research, Cologne, Germany)^50,51^, based on mutual information^52^, to spatially align the images and compensate for minor head motion. Head movement was quantified by taking the square root of the sum of movement in millimetres squared in *x*, *y*, and *z* directions from the last PET frame relative to the reference frame. Prior to pharmacokinetic analysis, volumes of interest (cerebellum and striatum) were generated automatically using PVElab (v2.3; Neurobiology Research Unit, Copenhagen, Denmark)^53,54^ based on a maximum probability atlas^55^. In addition, SPM12 (Wellcome Centre for Human Neuroimaging, London, UK) was used to segment the MRI image into grey (GM) and white matter (WM). Subsequently, the GM cerebellum was used as a reference region to calculate the influx constant *k*_i_^cer^ (min^-1^; from here on labelled as *k*_i_^cer^) as a measure of dopamine synthesis capacity using reference Patlak graphical analysis^56^ as implemented in PPET (Amsterdam UMC, Amsterdam, The Netherlands)^57^. PET frames acquired between minutes 25 and 90 were used for linear fit, resulting in a whole-brain parametric image. From this parametric image, *k*_i_^cer^ for the GM striatum was extracted.

The functional striatal sub-regions (associative, limbic, and sensorimotor), defined in the Oxford-GSK-Imanova brain atlas^58^, and a standard Montreal Neurological Imaging (MNI) brain template were extracted from FSLeyes v0.3 in FSL (v6.0; Analysis Group, Oxford, United Kingdom)^59,60^. Using Vinci, the MNI-template was warped to match the participant MRI using a non-linear affine transformation. The resulting transformation matrix was applied to the striatal sub-regions, thereby warping them from standard- to subject-space. From the parametric image, we extracted GM *k*_i_^cer^ values for voxels with at least 60% probability of belonging to the functional striatal sub-regions^61^.

We conducted several additional analyses to ensure the validity of the methods that we used (reported in Supplements 3.1–3.6). First, we calculated a whole-striatum *k*_i_^cer^ value obtained from the three sub-regions weighted by their respective volumes and compared this to the *k*_i_^cer^ value for the striatum obtained from the maximum probability atlas. Second, we repeated our main analyses with *k*_i_^cer^ values extracted from the combined GM and WM tissue, since previous studies examining dopamine synthesis capacity did not always separate these tissues. Third, in the analyses with the striatal sub-regions, we additionally investigated a stricter threshold of at least 90% probability. Fourth, we compared the mean standardized uptake values (SUV) in GM cerebellum in ASD and controls, to examine whether non-specific uptake of [^18^F]-FDOPA differed between groups. Finally, we repeated the main analyses for the two PET/CT-scanners separately.

### Statistical analyses

Statistical analyses were conducted using SPSS v26. A two-tailed alpha of α = 0.05 was used to evaluate statistical significance. Statistical test assumptions were adequately met, unless stated otherwise. We had few missing data on the measures of social defeat (less than 0.25% of total responses on any questionnaire). Since imputation of these missing data did not change the outcomes of the study, we report the results without these data imputed.

For the primary analyses, we compared the striatal *k*_i_^cer^ between ASD and controls using ANCOVA. We examined the relationship between striatal *k*_i_^cer^ and loneliness within ASD by conducting linear regression analysis. Analyses were adjusted for age, sex, smoking-status, and scanner-type (i.e., Biograph Horizon or Vereos) (for unadjusted results, see Supplement 4.1).

In the secondary analyses, group differences in social defeat were examined using independent-samples *t*-tests or *χ*^2^-tests. We report these results without adjusting for covariates, but regression analyses adjusted for age, sex, and smoking-status are reported in Supplement 4.2. The measures of childhood trauma and total bullying duration violated assumptions of normality and equality of variances, which we resolved by computing bootstrapped *p*-values^62,63^. Furthermore, we conducted regression analyses to examine relationships between measures of social defeat (besides loneliness) and striatal *k*_i_^cer^. Finally, we conducted the previously-described analyses using the *k*_i_^cer^ in the three striatal sub-regions (rather than whole striatum) as dependent variables.

## Results

### Participants

Forty-five adults with ASD and 24 controls completed the study. For technical reasons, we were unable to use the data of three participants (*n* = 1 ASD, *n* = 2 controls). Thus, the final sample included 44 adults with ASD and 22 controls. Table 1 shows the characteristics of the two samples. Average head movement was comparable in ASD and controls. We included 3 participants with substantial head movement (>5 mm; 1 control and 2 ASD participants), as we excluded deviating frames during parametric analysis and the *k*_i_^cer^ values of these participants fell within the range of values of the other participants. Moreover, excluding them did not alter the results.

**Table 1:** Sample characteristics of adults with autism spectrum disorder (ASD) and controls.

Variable	ASD (*n* = 44)	Controls (*n* = 22)
Male, No. (%)	28 (64%)	14 (64%)
Age in years, mean (SD)	23.74 (2.64)	23.47 (2.48)
Smoker, No. (%)	2 (5%)	1 (5%)
Scanned on Vereos PET/CT-scanner, No. (%)	31 (70%)	13 (59%)
IQ, mean (SD)	103.75 (5.19)	105.05 (4.90)
Approximate injected [^18^F]-FDOPA dose in MBq, mean (SD)	161.55 (7.26)	157.24 (8.57)
Head movement in millimetres, mean (SD)	2.44 (2.78)	2.54 (1.67)

*SD* standard deviation, *IQ* intelligence quotient, *MBq* megabecquerel.

### Dopamine synthesis capacity in ASD and controls

**Fig. 1:**
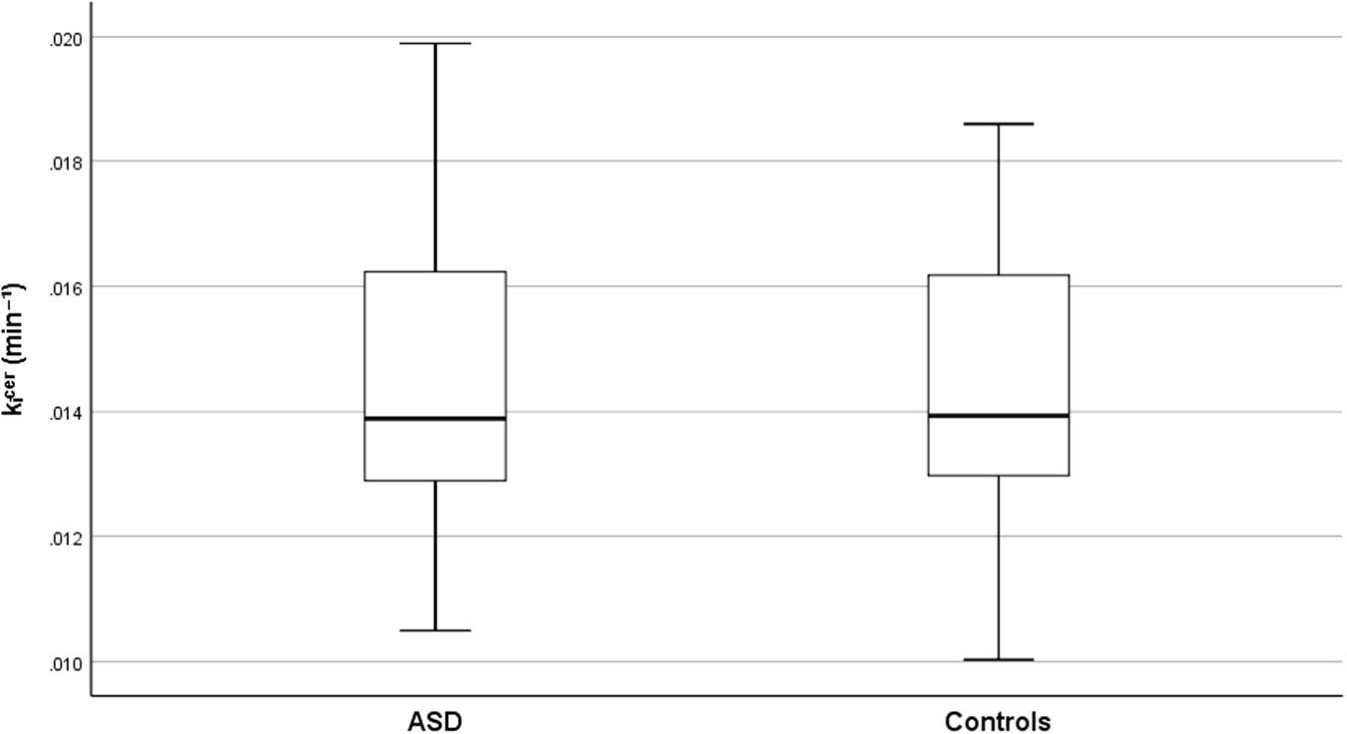
Striatal dopamine synthesis capacity in adults with autism spectrum disorder (ASD) and controls. Boxplots show the median, quartiles, and range of striatal presynaptic dopamine synthesis capacity (*k*_i_^cer^min^-1^).

**Fig. 2:**
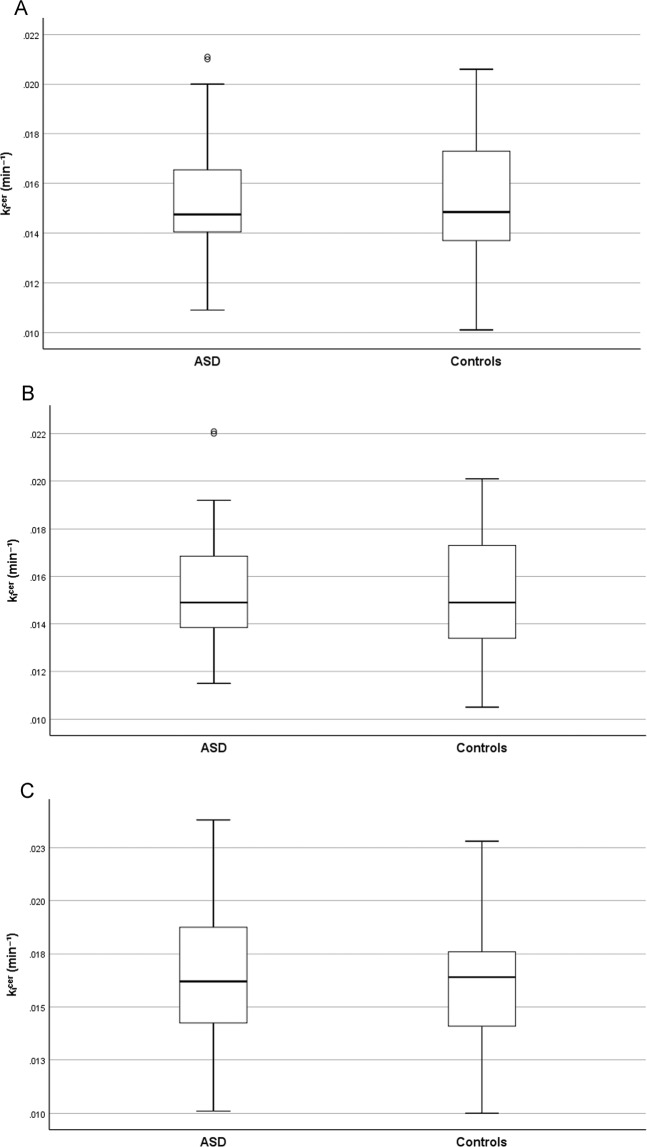
Dopamine synthesis capacity in striatal sub-regions in adults with autism spectrum disorder (ASD) and controls. Boxplots show the median, quartiles, and range of presynaptic dopamine synthesis capacity (*k*_i_^cer^min^-1^) in the **A** associative, **B** limbic, and **C** sensorimotor striatum.

Figure 1 shows the dopamine synthesis capacity in the striatum of adults with ASD and controls. In contrast to hypothesis 1a, we found no significant differences in dopamine synthesis capacity in ASD (*M* = 0.0145, *SD* = 0.0023) and controls (*M* = 0.0143, *SD* = 0.0024) in the striatum after adjusting for covariates (*F*_1,60_ = 0.026, *p* = 0.87). Moreover, as shown in Fig. 2, secondary analyses showed no significant group differences in dopamine synthesis capacity in the associative (ASD: *M* = 0.0155, *SD* = 0.0024; controls: *M* = 0.0155, *SD* = 0.0027; *F*_1,60_ = 0.003, *p* = 0.96), limbic (ASD: *M* = 0.0152, *SD* = 0.0026; controls: *M* = 0.0151, *SD* = 0.0028; *F*_1,60_ = 0.000, *p* > 0.99), or sensorimotor (ASD: *M* = 0.0165, *SD* = 0.030; controls: *M* = 0.0166, *SD* = 0.0032; *F*_1,60_ = 0.011, *p* = 0.92) striatal sub-regions after adjusting for covariates.

### Association between loneliness and dopamine synthesis capacity in ASD

**Fig. 3:**
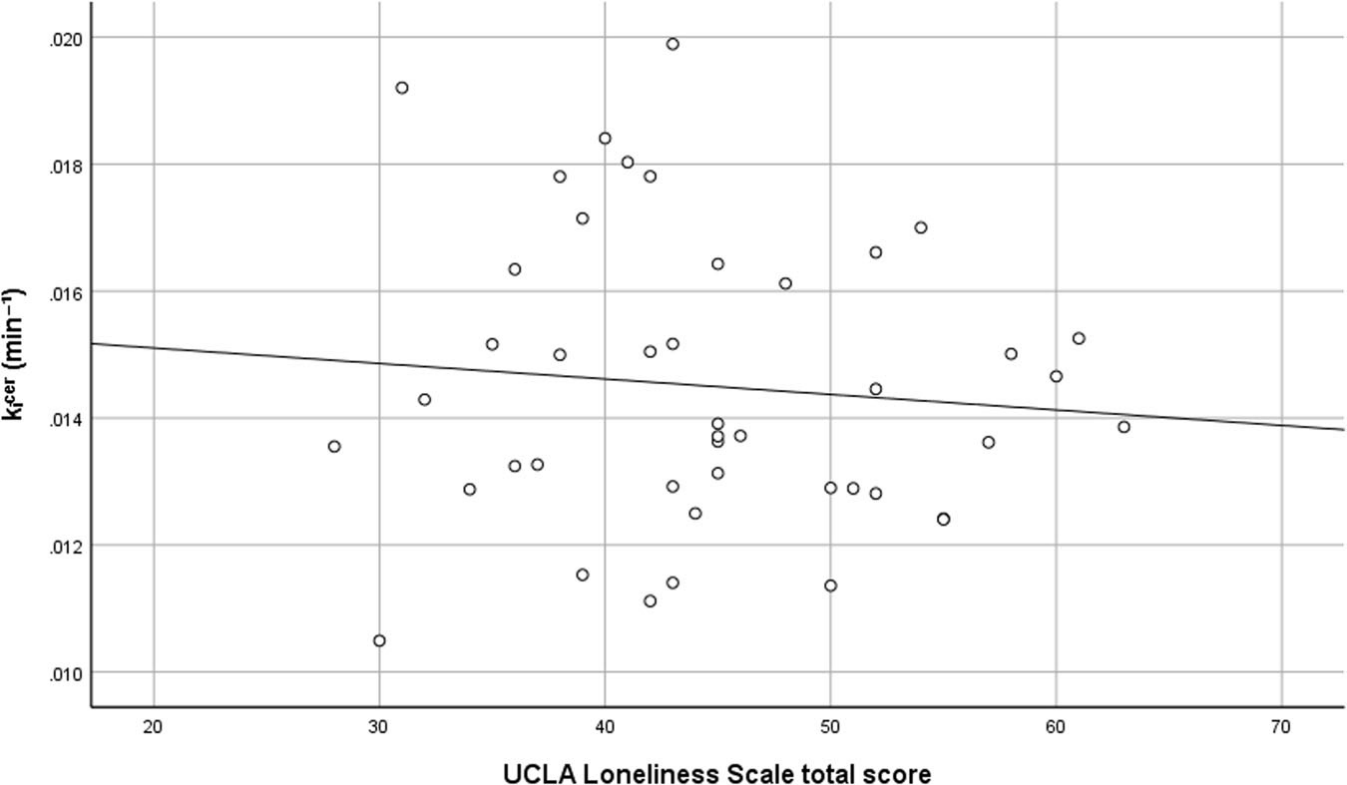
Association between loneliness and striatal dopamine synthesis capacity in adults with autism spectrum disorder (ASD). Scatterplot shows the unadjusted relationship between total scores on the UCLA Loneliness Scale and presynaptic dopamine synthesis capacity (*k*_i_^cer^min^-1^) in the whole striatum.

In contrast to hypothesis 1b, among adults with ASD there was no statistically significant association between loneliness and dopamine synthesis capacity in the striatum after adjusting for covariates (*β* = 0.01, *p* = 0.96) (see Fig. 3). Furthermore, secondary analyses showed no significant associations between loneliness and dopamine synthesis capacity in the associative (*β* = −0.03, *p* = 0.87), limbic (*β* = −0.01, *p* = 0.93), or sensorimotor (*β* = 0.01, *p* = 0.97) striatal sub-regions after adjusting for covariates.

### Secondary analyses with social defeat

Adults with ASD reported more loneliness, more experiences of being ostracized, more and longer-lasting episodes of bullying victimization, smaller social networks, and more traumatic experiences in childhood than controls (see Table 2). Both samples reported a similar need to belong, but adults with ASD reported a lower availability of interpersonal support.

**Table 2:** Measures of social defeat in adults with autism spectrum disorder (ASD) and controls.

Variable	ASD (*n* = 44)	Controls (*n* = 22)	*p*-value
UCLA Loneliness Scale, mean (SD)	44.66 (8.65)	32.14 (5.77)	*p* < 0.001^a^
OES, mean (SD)	29.73 (5.76)	21.86 (4.35)	*p* < 0.001^a^
Bullied (yes/no), No. (%)	36 (82%)	11 (50%)	*p* = 0.01^d^
Bullying total duration in months, mean (SD)	48.57 (40.57)	11.32 (18.63)	*p* < 0.001^a,b,c^
LSNS, mean (SD)	15.89 (5.34)	21.36 (3.13)	*p* < 0.001^a,b^
CTQ, mean (SD)	38.70 (12.29)	30.32 (4.65)	*p* < 0.001^a,b,c^
NBS, mean (SD)	31.36 (6.43)	31.45 (4.62)	*p* = 0.95^a,b^
ISEL, mean (SD)	119.25 (16.13)	137.45 (7.79)	*p* < 0.001^a,b^

*SD* standard deviation, *OES* Ostracism Experience Scale, *LSNS* Lubben Social Network Scale, *CTQ* Childhood Trauma questionnaire, *NBS* Need to Belong Scale, *ISEL* Interpersonal Support Evaluation List.

^a^Independent-samples *t*-test.

^b^Equal variances not assumed.

^c^Estimated with 10 000 bootstrapping samples.

^d^*χ*^2^-test.

The relations between the secondary measures of social defeat and striatal dopamine synthesis capacity in adults with ASD are shown in Supplement 4.3. We found no significant association between the measures of social defeat with dopamine synthesis capacity in the striatum or any of its sub-regions after adjusting for covariates. A possible exception to this was childhood trauma (see Supplement 4.4), which did show a significant positive association with striatal dopamine synthesis capacity (*β* = 0.32, *p* = 0.05). However, this association was largely driven by one outlier (after excluding, *β* = 0.20, *p* = 0.25) and became non-significant after adjusting the *p*-value for multiple testing.

Notably, contrary to our expectations, exploratory post-hoc analyses in controls showed a significant negative association between childhood trauma and striatal dopamine synthesis capacity (Supplement 4.5 and 4.6). On the other hand, the relationship between social support and dopamine synthesis capacity was negative in controls with a high need to belong (but positive in those with a low need to belong; Supplement 4.7).

### Associations with symptoms

Adults with ASD (*M* = 3.13, *SD* = 1.93) reported more psychotic symptoms on the PQ-16 than controls (*M* = 1.06, *SD* = 0.97). However, these symptoms were not significantly associated with dopamine synthesis capacity in the striatum in ASD (*β* = −0.05, *p* = 0.81) or in controls (*β* = −0.26, *p* = 0.30) after adjusting for covariates. Results were also non-significant for the striatal sub-regions (see Supplement 4.8).

Exploratory analyses showed no statistically significant associations between dopamine synthesis capacity and depressed mood or anxiety (see Supplement 4.9).

### Sensitivity analyses

As shown throughout Supplement 3, *k*_i_^cer^ values somewhat differed depending on the image processing procedure and/or PET/CT-scanner that was used, but the results of the analyses were comparable and conclusions remained unchanged. Non-specific uptake of [^18^F]-FDOPA in the cerebellum was similar in ASD and controls.

## Discussion

We used PET/CT to examine striatal dopamine synthesis capacity in unmedicated, non-psychotic adults with ASD and its association with measures of social defeat. Contrary to our expectations, adults with ASD did not have a greater presynaptic dopamine synthesis capacity in the striatum or any of its sub-regions relative to controls. Furthermore, among adults with ASD we found no significant association between striatal dopamine synthesis capacity and loneliness or other measures of social defeat.

Our findings do not support previous ideas about the involvement of presynaptic dopamine signalling in ASD^8^. However, dopamine synthesis capacity is only one aspect of dopamine signalling and other studies in ASD have found local alterations in dopamine transporter binding^64^ and dopamine receptor density^65^. Moreover, it is well-established that ASD is heterogeneous^66^, and as a result, deviations in dopamine synthesis might be present only in a subgroup of individuals. ASD adults with a low IQ, who used medication, and/or who had substantial psychotic symptoms were excluded from our study, and it would be interesting to examine whether abnormalities in dopamine synthesis capacity are present among them. Future studies can further elucidate whether alterations in dopamine functioning are involved in ASD.

The social defeat hypothesis of schizophrenia, which predicts that non-psychotic adults with ASD show an increase in dopamine synthesis capacity and that this capacity is associated with measures of social defeat^19^, was not supported. This might be explained as follows. First, the social defeat hypothesis might be incorrect. However, studies that examined striatal dopamine synthesis capacity and/or release in immigrants^23^, individuals with severe hearing impairment^20^, and individuals exposed to childhood trauma^21,22^ supported this hypothesis. Furthermore, experiments reported increased dopamine release in the nucleus accumbens of (sub)chronically defeated rodents^67^. In contrast, in line with post-hoc analyses with reference to childhood trauma in our control group, two recent studies observed negative associations between different types of social adversity and dopamine synthesis capacity^68^ or amphetamine-induced striatal dopamine release^69^. One possibility is that social defeat only increases dopamine functioning in certain individuals and that, for example, individuals with ASD respond neurochemically different to defeat than other risk groups. Nevertheless, given the high rates of social defeat in adults with ASD and the absence of a relationship with presynaptic dopamine, one can conclude from this study that social defeat per se does not *necessarily* upregulate dopamine synthesis capacity.

Second, dopamine sensitization might have been demonstrated if presynaptic dopamine release had been examined, for instance after a challenge with amphetamine. Since knowledge about the relationship between presynaptic dopamine synthesis capacity and amphetamine-induced dopamine release is limited, it is difficult to predict the outcome of this challenge^70^. To the best of our knowledge, presynaptic dopamine release has not yet been examined in ASD and it might be worthwhile to do so. It should be performed with great care, however, since inducing dopamine release might precipitate psychotic symptoms.

This study has several strengths. First, this is by far the largest PET/CT-study to examine dopamine synthesis capacity in ASD and the first to examine an association with possible risk factors. Given the (for PET/CT studies) large, a priori determined sample size, it is highly unlikely that the negative findings are due to a lack of statistical power. Second, we had strict inclusion measures, which might limit the generalizability of the findings to the entire ASD population but does reduce the influence of external factors such as (dopaminergic) medication use. Third, in addition to our main analysis with loneliness, we used multiple measures to assess social defeat, which consistently showed an absence of relation with striatal dopamine functioning.

The study also has several limitations. First, the study was cross-sectional. To understand how dopaminergic abnormalities arise, longitudinal studies are necessary. Second, we relied on self-report measures of social defeat, which may be prone to several types of bias. Third, PET/CT-scans were acquired on two different PET/CT systems. However, reconstruction parameters for the two scanners were harmonized as much as possible and scanner-type was added as a covariate to the analyses. Moreover, conclusions did not change when we conducted the main analyses for the two PET/CT-scanners separately.

In conclusion, non-psychotic, unmedicated adults with ASD do not show a significant increase in striatal dopamine synthesis capacity, and this capacity is not associated with measures of social defeat.

## Supplementary information

Supplementary information
