# Supplementary material for: Striatal dopamine synthesis capacity in autism spectrum disorder and its relation with social defeat: an [18F]-FDOPA PET/CT study
Source: Transl Psychiatry. 2021 Jan 13;11:47. doi: 10.1038/s41398-020-01174-w (PMC7806928; doi:10.1038/s41398-020-01174-w)
Supplement: Supplementary file 1 — Supplementary information [file 41398_2020_1174_MOESM1_ESM.docx]

**Supplementary Information**

**Table of contents**

[**Supplement 1. Full list of in- and exclusion criteria** 2](#_Toc57025198)

[**Supplement 2. Description of measures** 3](#_Toc57025199)

[**Supplement 3. Control analyses** 5](#_Toc57025200)

[**Supplement 4. Secondary analyses** 11](#_Toc57025201)

[**Supplementary References** 21](#_Toc57025202)

# **Supplement 1. Full list of in- and exclusion criteria**

**Inclusion criteria**

1. DSM-5 diagnosis of autism spectrum disorder (ASD; only for the ASD sample).
2. Age: 18-30 years.

**Exclusion criteria**

1. DSM-5 diagnosis of ASD (controls only).
2. Autism spectrum disorder due to a known organic disorder (“Syndromal ASD”, e.g., due to Fragile X syndrome, Klinefelter syndrome, 22q11 deletion syndrome).
3. Neurological disorder (e.g., epilepsy) or evidence of brain damage.
4. History of meningitis.
5. IQ<85.
6. Non-affective psychotic disorder or bipolar disorder (DSM-5: 297.1, 298.8, 295.40, 295.90, 295.70, 292.9, 291.9, 293.81, 293.82, 293.89, 298.9, 296.89, 303.13, 293.83, 296.89, 296.80, 296.4x, 296.5x, 296.7).
7. Social exclusion due to other causes than ASD, such as visible ethnic minority status, serious physical disability, serious visual or hearing impairment, at discretion of the researcher.

*Exclusion criteria related to alcohol, soft/hard-drugs, medicinal drugs:*

1. Current use of drugs (XTC, cocaine, etc.). Use of cannabis was allowed, but should have been stopped at least one month before the study. Cannabis abuse earlier in life was not allowed.
2. Alcohol- or drug abuse or dependence (DSM-5).
3. Use of an antipsychotic (ever) if prescribed for a psychotic disorder, as a former psychotic disorder is an exclusion criterion. Occasionally, antipsychotics are prescribed against e.g. anxiety or aggression. In these cases:
   1. Incidental former use of antipsychotic was allowed, if the last use had been more than a year ago.
   2. Regular former use of antipsychotic was allowed, if the last use had been more than two years ago.
   3. Antipsychotic formerly administered as depot medication was allowed, if the last injection had been more than two years ago.
4. Use of the antipsychotic quetiapine (ever), if prescribed in relation to a psychotic disorder. However, quetiapine is often prescribed against sleep difficulties and has a low affinity to dopamine receptors^1^. In these cases:
   1. Consumption was allowed if previously consumed in a low dose (≤50mg), but last use had been more than 3 months ago.
   2. Consumption was allowed if previously consumed in a high dose (>50mg), but last use had been more than 6 months ago.
5. Use of medication usually prescribed for attention-deficit/hyperactivity disorder (ADHD) (e.g., methylphenidate). Individuals who had stopped using these drugs for at least one year could participate in the study.
6. Use of benzodiazepine or promethazine, unless last use had been more than 1 month ago.
7. Use of other psychotropic drugs. Individuals who had stopped using the drugs for at least 3 months could participate in the study.

*Exclusion criteria directly related to MRI- and PET/CT-scanning:*

1. Smoking during the period of three hours prior to the PET/CT-scan and eating or drinking (except water) during the period of six hours prior to the PET/CT-scan.
2. Participation in a scientific examination where radiation was used, in the last year before participation.
3. Positive urine drug screen on the day of the PET/CT-scan. Participants were tested on cannabis, amphetamine, cocaine, and opiates.
4. In women: positive pregnancy test on the day of the PET/CT-scan and/or lactation.
5. Metal objects in or around the body.

Note that we did not ask participants to change their behavior in order to participate in this study. For example, we did not ask them to stop using medication for a month so that they could participate. Instead, if participants did not comply with the in- or exclusion criteria, we excluded them from current and future participation.

# **Supplement 2. Description of measures**

**Primary outcome**

*UCLA Loneliness Scale*: We used the UCLA Loneliness Scale^2^ to measure loneliness. We selected this questionnaire as the primary measure of social defeat, since it reflects a lack of social participation as well as the subjective negative experience of this, in line with the definition and previous studies of social defeat^3, 4^. The questionnaire consists of 20 items (e.g., “How often do you feel that you lack companionship?”), which are scored between 1 (Never) and 4 (Always). After reverse-coding, total scores range from 20 to 80, with higher scores indicating more feelings of loneliness.

**Secondary outcomes**

In addition to measuring loneliness, we assessed other measures of social defeat. Furthermore, we assessed the presence of psychotic symptoms. The following instruments were used:

*Ostracism Experience Scale (OES)*: The OES^5^ measures the extent to which participants feel excluded or ignored (i.e., ostracized). The questionnaire consists of 11 items (e.g., “In general, others ignore me during conversation”), which are scored between 1 (Never) and 5 (Always). Total scores range from 11 to 55, with higher scores indicating more experiences of being ostracized.

*Bullying Interview*: We conducted a semi-structured interview based on the definition of bullying proposed by Olweus^6^, to assess whether participants had been the victim of bullying prior to age 17. This definition was given as follows: “We say a child is being bullied when another child, or several other children say mean and hurtful things or make fun of him or her or call him or her mean and hurtful names; completely ignore or exclude him or her from their group of friends or leave him or her out of things on purpose; hit, kick, push, shove around, or lock him or her inside a room; tell lies or spread false rumors about him or her or send mean notes and try to make other children dislike him or her; and other hurtful things like that. When we talk about bullying, these things happen repeatedly, and it is difficult for the child being bullied to defend himself or herself. We also call it bullying, when a child is teased repeatedly in a mean and hurtful way. But we don’t call it bullying when the teasing is done in a friendly and playful way. Also, it is not bullying when two children of about equal strength or power argue or fight.”. After giving this definition, we asked questions about the frequency (i.e., number of bullying episodes), chronicity (i.e., duration of each bullying episode), severity, and age of bullying victimization. We used a binarized outcome of bullying victimization (bullied yes/no), as well as the total duration in months of the bullying episode(s).

*Lubben Social Network Scale (LSNS)*: The 6-item LSNS^7^ assesses social network size. The questionnaire asks about the number of family and friends that participants regularly engage with or can engage with if they need help or support (e.g., "How many relatives do you see or hear from at least once a month?"). Scores range from 6 to 36. Higher scores indicate the availability of a greater social network.

*Childhood Trauma Questionnaire (CTQ)*: The 24-item version of the CTQ^8^ is a retrospective self-report questionnaire that assesses childhood maltreatment occurring before age 17. Items contain questions about five domains of maltreatment (physical, emotional, and sexual abuse, and physical and emotional neglect; e.g., “When I was growing up, I thought that my parents wished I had never been born.”), which are scored between 1 (Never true) and 5 (Very often true). All subscales consist of five items, except for the sexual abuse subscale from which one item was removed in Dutch validation studies^9, 10^. In this study total scores (between 24 and 120) were used, with greater scores indicating more experiences of childhood trauma.

*Need to Belong Scale (NBS)*: The NBS^11^ assesses the need for social acceptance and belonging. The questionnaire consists of 10 items (e.g., “I want other people to accept me.”), which are scored between 1 (Not at all) and 5 (Extremely). After reverse-coding, total scores range from 10 to 50, with higher scores indicating a greater need to belong.

*Interpersonal Support Evaluation List (ISEL)*: The ISEL^12^ measures the extent to which participants perceive that social support is available. The questionnaire consists of 40 items (e.g., "If I decide one afternoon that I would like to go to a movie that evening, I could easily find someone to go with me."), which are scored between 1 and 4. Total scores range from 40 to 160. Higher scores indicate a greater availability of social support.

*Comprehensive Assessment of At-Risk Mental States (CAARMS)*. The CAARMS^13, 14^ is a semi-structured interview, which assesses the presence of positive psychotic symptoms. The interview is most often used to classify participants as being at a clinically high risk of developing a psychotic disorder. This classification is made in case of past-year reduced social functioning, combined with the presence of attenuated psychotic symptoms or the presence of a psychotic disorder in first-degree relatives.

*Prodromal Questionnaire-16 (PQ-16)*: The PQ-16^14, 15^ was used to assess self-reported psychotic symptoms. The questionnaire consists of 16 items (e.g., “I have heard things other people can't hear like voices of people whispering or talking”), which are dichotomously scored (False=0 or True=1). Total scores range from 0 to 16.

# **Supplement 3. Control analyses**

Here we show the results of additional analyses that we conducted to compare our calculations of striatal *k*_i_^cer^ values to other methods. Supplement 3.1 shows the whole-striatum *k*_i_^cer^ values calculated with different methods. In Supplement 3.2 we show that these values are highly correlated. In Supplement 3.3 we show that the conclusions of the main findings remain the same, regardless of which method is used. In Supplement 3.4, we show that the *k*_i_^cer^ values obtained from striatal sub-regions calculated with a strict 90% threshold, give the same results as those with a 60% threshold. In Supplement 3.5, we show that mean standardized uptake values (SUV), corrected for body weight, in cerebellar gray matter are similar in ASD and controls. Finally, we show that the results of the main analyses remain unchanged when examined for the two PET/CT scanners separately (Supplement 3.6).

| **Supplement 3.1. Presynaptic dopamine synthesis capacity (*k*_i_^cer^) for the whole striatum, calculated with different methods.** | | | |
| --- | --- | --- | --- |
| **Whole striatum *k*_i_^cer^ calculated from** | **Sample** | **GM (mean [SD], range)** | **GM+WM (mean [SD], range)** |
| Probabilistic atlas^1^ | ASD | 0.0145 (0.0023)^3^, 0.0105–0.0199 | 0.0135 (0.0020), 0.0098–0.0179 |
|  | Controls | 0.0143 (0.0024)^3^, 0.0100–0.0186 | 0.0134 (0.0023), 0.0093–0.0173 |
| Combined striatal sub-regions from FSL^2^ (60% threshold) | ASD | 0.0155 (0.0024), 0.0110–0.0214 | 0.0153 (0.0024), 0.0109–0.0210 |
|  | Controls | 0.0155 (0.0027), 0.0102–0.0204 | 0.0153 (0.0027), 0.0102–0.0204 |
| Combined striatal sub-regions from FSL^2^ (90% threshold) | ASD | 0.0167 (0.0027), 0.0119–0.0238 | 0.0167 (0.0027), 0.0118–0.0237 |
|  | Controls | 0.0167 (0.0029), 0.0112–0.0224 | 0.0167 (0.0029), 0.0112–0.0224 |
| GM, gray matter; WM, white matter; SD, standard deviation; ASD, autism spectrum disorder.  ^1^ See: Hammers et al.^16^  ^2^ See: Tziortzi et al.^17^, Jenkinson et al.^18^, McCarthy^19^  ^3^ As reported in the main article. | | | |

| **Supplement 3.2. Pearson correlations between *k*_i_^cer^ values for the whole striatum in the entire sample (ASD+controls), calculated with different methods.** | | | | | | |
| --- | --- | --- | --- | --- | --- | --- |
| **Whole striatum *k*_i_^cer^ calculated from:** | **Probabilistic atlas^1^ (GM)** | **Probabilistic atlas^1^ (GM+WM)** | **Combined striatal sub-regions^2^ (60%; GM)** | **Combined striatal sub-regions^2^ (60%; GM+WM)** | **Combined striatal sub-regions^2^ (90%; GM)** | **Combined striatal sub-regions^2^ (90%; GM+WM)** |
| Probabilistic atlas^1^ (GM) | 1 |  |  |  |  |  |
| Probabilistic atlas^1^ (GM+WM) | 0.986 | 1 |  |  |  |  |
| Combined striatal sub-regions^2^ (60%; GM) | 0.969 | 0.953 | 1 |  |  |  |
| Combined striatal sub-regions^2^ (60%; GM+WM) | 0.966 | 0.955 | 0.998 | 1 |  |  |
| Combined striatal sub-regions^2^ (90%; GM) | 0.962 | 0.944 | 0.996 | 0.995 | 1 |  |
| Combined striatal sub-regions^2^ (90%; GM+WM) | 0.960 | 0.945 | 0.995 | 0.996 | 0.999 | 1 |
| ASD, autism spectrum disorder; GM, gray matter; WM, white matter.  ^1^ See: Hammers et al.^16^  ^2^ See: Tziortzi et al.^17^, Jenkinson et al.^18^, McCarthy^19^ | | | | | | |

**Supplement 3.3. Main analyses with alternative calculations of whole-striatum *k*_i_^cer^**

Regardless of the method used to calculate the whole-striatum *k*_i_^cer^, the *k*_i_^cer^ did not differ between adults with ASD and controls (lowest *p*=0.91). Moreover, none of the *k*_i_^cer^ values were associated with loneliness in adults with ASD (lowest *p*=0.24).

**Supplement 3.4. *k*_i_^cer^ in striatal sub-regions with 90% threshold**

When we used a stricter 90% threshold to calculate the *k*_i_^cer^ values in the striatal sub-regions, we still found no significant difference in *k*_i_^cer^ values in the associative, limbic, or sensorimotor striatum between adults with ASD and controls (lowest *p*=0.77) and none of these values were associated with loneliness in adults with ASD (lowest *p*=0.21).

| **Supplement 3.5. Mean standardized uptake values (SUV) of [^18^F]-FDOPA in gray matter cerebellum in minutes post-injection in adults with autism spectrum disorder (ASD) and controls.** |
| --- |
| 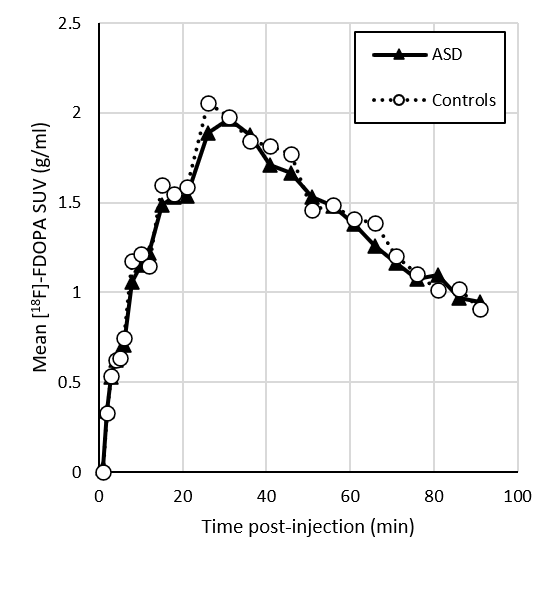 |

**Supplement 3.6. Results of the main analyses for the two PET/CT scanners separately.**

When we restricted the analyses to data collected on the Siemens Biograph Horizon, we found no statistically significant difference in whole-striatal *k*_i_^cer^ between ASD (*n* = 13, *M* = 0.0143, *SD* = 0.0026) and controls (*n* = 9, *M* = 0.0139, *SD* = 0.0026) (*F*_1,20_ = 0.09, *p* = 0.77; adjusted for age, sex, smoking: *F*_1,17_ = 0.46, *p* = 0.51). On average, the whole-striatal *k*_i_^cer^ collected on the Philips Vereos was somewhat higher in ASD (*n* = 31, *M* = 0.0146, *SD* = 0.0022) and in controls (*n* = 13, *M* = 0.0146, *SD* = 0.0024), but on this scanner we also found no significant group differences (*F*_1,42_ = 0.001, *p* = 0.97; adjusted for age, sex, smoking: *F*_1,39_ = 0.08, *p* = 0.77).

Furthermore, we found no association between loneliness and whole-striatum *k*_i_^cer^ in ASD when only looking at data collected on the Siemens Biograph Horizon (*n* = 13; *β* = 0.03, *p* = 0.92; adjusted for age, sex, smoking: *β* = 0.24, *p* = 0.34) or Philips Vereos (*n* = 31; *β* = -0.13, *p* = 0.48; adjusted for age, sex, smoking: *β* = -0.15, *p* = 0.47).

# **Supplement 4. Secondary analyses**

On the next pages we show the results of several secondary analyses. We first show the difference in striatal *k*_i_^cer^ between ASD and controls, and the association between striatal *k*_i_^cer^ and loneliness in ASD, without adjusting for covariates (Supplement 4.1). Next, we report the differences in measures of social defeat between ASD and controls after adjusting for age, sex, and smoking status, using (logistic) regression analyses (Supplement 4.2). We then show associations between striatal *k*_i_^cer^ and other measures of social defeat in ASD (Supplement 4.3 and 4.4) and controls (Supplements 4.5, 4.6, and 4.7). We then show associations between measures of psychosis (Supplement 4.8) and depression and anxiety (Supplement 4.9) and striatal *k*_i_^cer^ in ASD and controls.

| **Supplement 4.1. Striatal *k*_i_^cer^ in adults with autism spectrum disorder (ASD) and controls and its association with loneliness in ASD, without adjusting for covariates.** | |
| --- | --- |
| **Analysis** | **Test statistic** |
| Difference in *k*_i_^cer^ between ASD and controls |  |
| Whole-striatum *k*_i_^cer^ | *F*_1,64_=0.069, *p*=0.79 |
| Associative striatum *k*_i_^cer^ | *F*_1,64_=0.000, *p*>0.99 |
| Limbic striatum *k*_i_^cer^ | *F*_1,64_=0.026, *p*=0.87 |
| Sensorimotor striatum *k*_i_^cer^ | *F*_1,64_=0.000, *p*=0.99 |
| Association between *k*_i_^cer^ and loneliness in ASD |  |
| Whole-striatum *k*_i_^cer^ | *β*=-0.09, *p*=0.55 |
| Associative striatum *k*_i_^cer^ | *β*=-0.16, *p*=0.31 |
| Limbic striatum *k*_i_^cer^ | *β*=-0.14, *p*=0.37 |
| Sensorimotor striatum *k*_i_^cer^ | *β*=-0.10, *p*=0.50 |

| **Supplement 4.2. Differences in measures of social defeat between adults with autism spectrum disorder and controls after adjusting for age, sex, and smoking-status (yes/no).** | |
| --- | --- |
| **Variable** | **Standardized beta, *p*-value** |
| UCLA Loneliness Scale | *β*=0.60, *p*<0.001^1^ |
| OES | *β*=0.57, *p*<0.001^1^ |
| Bullied (yes/no) | OR=4.5, *p*=0.01^2^ |
| Bullying total duration in months | *β*=0.46, *p*<0.001^1^ |
| LSNS | *β*=-0.48, *p*<0.001^1^ |
| CTQ | *β*=0.36, *p*=0.003^1^ |
| NBS | *β*=0.003, *p*=0.98^1^ |
| ISEL | *β*=-0.52, *p*<0.001^1^ |
| OES, Ostracism Experience Scale; LSNS, Lubben Social Network Scale; CTQ, Childhood Trauma Questionnaire; NBS, Need to Belong Scale; ISEL, Interpersonal Support Evaluation List; OR, odds ratio.  ^1^ Multivariable linear regression analysis.  ^2^ Multivariable logistic regression analysis. | |

| **Supplement 4.3. Associations between measures of social defeat and striatal *k*_i_^cer^ in adults with autism spectrum disorder (ASD), before and after adjusting for covariates.** | | | | | |
| --- | --- | --- | --- | --- | --- |
|  |  | **Striatal region (*k*_i_^cer^)** | | | |
| **Variable** | **Covariates^1^** | Whole striatum | Associative striatum | Limbic striatum | Sensorimotor striatum |
| OES | Non-adjusted | *β*=-0.19, *p*=0.23 | *β*=-0.24, *p*=0.12 | *β*=-0.25, *p*=0.11 | *β*=-0.13, *p*=0.41 |
|  | Adjusted | *β*=-0.12, *p*=0.51 | *β*=-0.12, *p*=0.48 | *β*=-0.16, *p*=0.34 | *β*=0.01, *p*=0.94 |
| Bullied (yes/no) | Non-adjusted | *β*=-0.16, *p*=0.30 | *β*=-0.20, *p*=0.19 | *β*=-0.16, *p*=0.30 | *β*=-0.22, *p*=0.14 |
|  | Adjusted | *β*=-0.12, *p*=0.46 | *β*=-0.13, *p*=0.40 | *β*=-0.10, *p*=0.52 | *β*=-0.16, *p*=0.30 |
| Bullying total duration in months | Non-adjusted | *β*=-0.15, *p*=0.32 | *β*=-0.11, *p*=0.49 | *β*=-0.16, *p*=0.29 | *β*=-0.05, *p*=0.77 |
|  | Adjusted | *β*=-0.18, *p*=0.26 | *β*=-0.14, *p*=0.37 | *β*=-0.17, *p*=0.27 | *β*=-0.10, *p*=0.50 |
| LSNS | Non-adjusted | *β*=-0.25, *p*=0.11 | *β*=-0.19, *p*=0.21 | *β*=-0.15, *p*=0.34 | *β*=-0.20, *p*=0.20 |
|  | Adjusted | *β*=-0.31, *p*=0.05 | *β*=-0.24, *p*=0.12 | *β*=-0.23, *p*=0.14 | *β*=-0.20, *p*=0.19 |
| CTQ | Non-adjusted | *β*=0.31, *p*=0.04* | *β*=0.33, *p*=0.03* | *β*=0.31, *p*=0.04* | *β*=0.27, *p*=0.07 |
|  | Adjusted | *β*=0.32, *p*=0.05* | *β*=0.31, *p*=0.05 | *β*=0.35, *p*=0.03* | *β*=0.17, *p*=0.27 |
| NBS*ISEL^2^ | Non-adjusted | *β*=0.18, *p*=0.30 | *β*=0.12, *p*=0.51 | *β*=0.21, *p*=0.23 | *β*=-0.04, *p*=0.83 |
|  | Adjusted | *β*=0.18, *p*=0.35 | *β*=0.08, *p*=0.66 | *β*=0.14, *p*=0.46 | *β*=-0.03, *p*=0.88 |
| OES, Ostracism Experience Scale; LSNS, Lubben Social Network Scale; CTQ, Childhood Trauma Questionnaire; NBS, Need to Belong Scale; ISEL, Interpersonal Support Evaluation List; NBS*ISEL, interaction between the two variables.  ^1^ Covariates: age, sex, smoking-status (yes/no), PET/CT-scanner type (Biograph Horizon or Vereos)  ^2^ Variables were centered prior to calculating their interaction effect.  * *p*<0.05 | | | | | |

| **Supplement 4.4. Unadjusted scatterplot of the relationship between total scores on the Childhood Trauma Questionnaire (CTQ) and presynaptic dopamine synthesis capacity (*k*_i_^cer^) in the whole striatum in adults with autism spectrum disorder.** |
| --- |
|  |

**
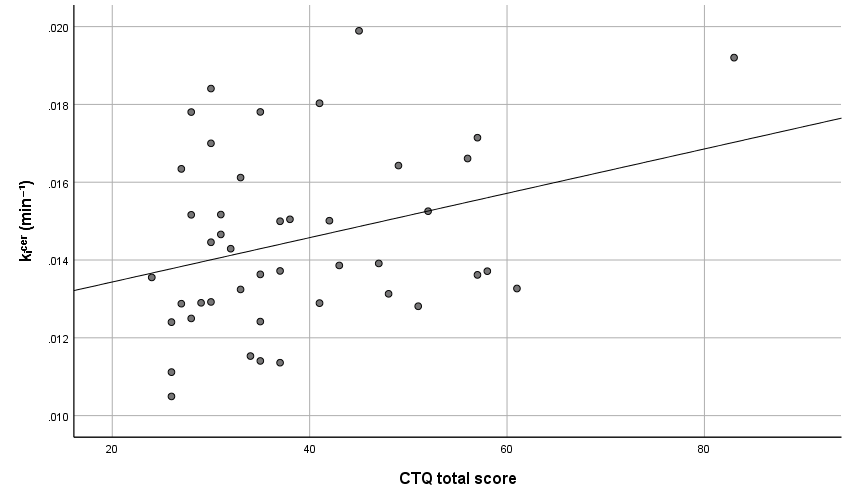
**

| **Supplement 4.5. Associations between measures of social defeat and striatal *k*_i_^cer^ in controls, before and after adjusting for covariates.** | | | | | |
| --- | --- | --- | --- | --- | --- |
|  |  | **Striatal region (*k*_i_^cer^)** | | | |
| **Variable** | **Covariates^1^** | Whole striatum | Associative striatum | Limbic striatum | Sensorimotor striatum |
| UCLA Loneliness Scale | Non-adjusted | *β*=-0.25, *p*=0.26 | *β*=-0.28, *p*=0.21 | *β*=-0.27, *p*=0.22 | *β*=-0.19, *p*=0.39 |
|  | Adjusted | *β*=-0.35, *p*=0.14 | *β*=-0.30, *p*=0.19 | *β*=-0.39, *p*=0.10 | *β*=-0.11, *p*=0.60 |
| OES | Non-adjusted | *β*=-0.22, *p*=0.32 | *β*=-0.20, *p*=0.38 | *β*=-0.24, *p*=0.28 | *β*=-0.07, *p*=0.76 |
|  | Adjusted | *β*=-0.29, *p*=0.19 | *β*=-0.28, *p*=0.19 | *β*=-0.30, *p*=0.18 | *β*=-0.13, *p*=0.49 |
| Bullied (yes/no) | Non-adjusted | *β*=-0.11, *p*=0.64 | *β*=-0.01, *p*=0.97 | *β*=-0.12, *p*=0.60 | *β*=0.11, *p*=0.61 |
|  | Adjusted | *β*=-0.10, *p*=0.72 | *β*=-0.14, *p*=0.62 | *β*=-0.07, *p*=0.80 | *β*=-0.15, *p*=0.54 |
| Bullying total duration in months | Non-adjusted | *β*=-0.31, *p*=0.16 | *β*=-0.31, *p*=0.17 | *β*=-0.32, *p*=0.15 | *β*=-0.27, *p*=0.23 |
|  | Adjusted | *β*=-0.20, *p*=0.41 | *β*=-0.24, *p*=0.29 | *β*=-0.21, *p*=0.39 | *β*=-0.28, *p*=0.16 |
| LSNS | Non-adjusted | *β*=0.16, *p*=0.49 | *β*=0.16, *p*=0.48 | *β*=0.15, *p*=0.49 | *β*=0.17, *p*=0.45 |
|  | Adjusted | *β*=0.17, *p*=0.45 | *β*=0.19, *p*=0.36 | *β*=0.16, *p*=0.49 | *β*=0.23, *p*=0.22 |
| CTQ | Non-adjusted | *β*=-0.38, *p*=0.08 | *β*=-0.36, *p*=0.10 | *β*=-0.37, *p*=0.09 | *β*=-0.27, *p*=0.23 |
|  | Adjusted | *β*=-0.51, *p*=0.03* | *β*=-0.50, *p*=0.03* | *β*=-0.48, *p*=0.05 | *β*=-0.41, *p*=0.04* |
| NBS*ISEL^2^ | Non-adjusted | *β*=-0.48, *p*=0.26 | *β*=-0.47, *p*=0.26 | *β*=-0.49, *p*=0.23 | *β*=-0.26, *p*=0.54 |
|  | Adjusted | *β*=-0.93, *p*=0.03* | *β*=-0.94, *p*=0.02* | *β*=-0.87, *p*=0.05 | *β*=-0.78, *p*=0.03* |
| OES, Ostracism Experience Scale; LSNS, Lubben Social Network Scale; CTQ, Childhood Trauma Questionnaire; NBS, Need to Belong Scale; ISEL, Interpersonal Support Evaluation List.  ^1^ Covariates: age, sex, smoking-status (yes/no), PET/CT-scanner type (Vereos or Biograph Horizon)  ^2^ Variables were centered prior to calculating their interaction effect.  * *p*<0.05 | | | | | |

| **Supplement 4.6. Unadjusted scatterplot of the relationship between total scores on the Childhood Trauma Questionnaire (CTQ) and presynaptic dopamine synthesis capacity (*k*_i_^cer^) in the whole striatum in controls.** |
| --- |
| 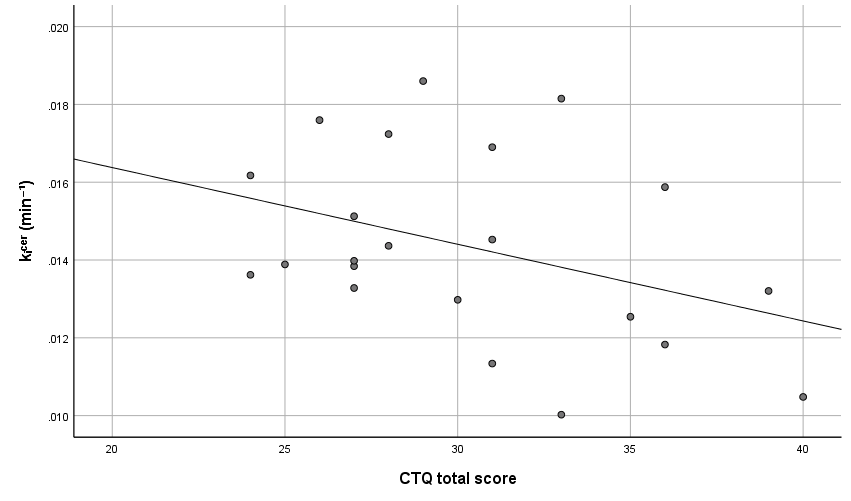 |

| **Supplement 4.7. Unadjusted scatterplot of the relationship between scores on the Interpersonal Support Evaluation List (ISEL) and presynaptic dopamine synthesis capacity (*k*_i_^cer^) in controls with lowest (*n*=7), medium (*n*=8), and highest (*n*=7) scores on the Need to Belong Scale (NBS).** |
| --- |
|  |
| 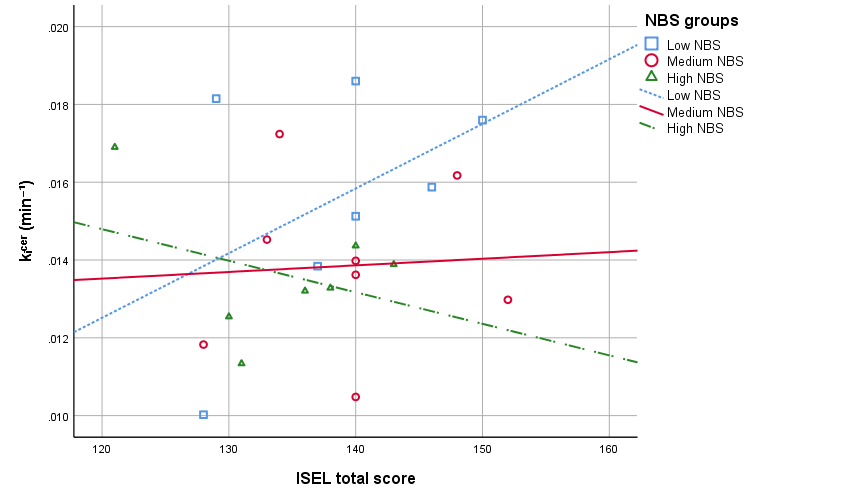 |

| **Supplement 4.8. Associations between striatal *k*_i_^cer^ and psychotic symptoms.** | | | | | |
| --- | --- | --- | --- | --- | --- |
|  |  | **Striatal region (*k*_i_^cer^)** | | | |
| **Analysis** | **Covariates^1^** | Whole striatum | Associative | Limbic | Sensorimotor |
| PQ-16 in ASD^5^ | Non-adjusted | *β*=-0.12, *p*=0.51 | *β*=-0.17, *p*=0.37 | *β*=-0.04, *p*=0.82 | *β*=-0.17, *p*=0.35 |
|  | Adjusted | *β*=-0.05, *p*=0.81^7^ | *β*=-0.10, *p*=0.61 | *β*=0.01, *p*=0.95 | *β*=-0.10, *p*=0.62 |
| PQ-16 in controls^6^ | Non-adjusted | *β*=-0.36, *p*=0.16 | *β*=-0.27, *p*=0.29 | *β*=-0.34, *p*=0.18 | *β*=-0.05, *p*=0.86 |
|  | Adjusted | *β*=-0.26, *p*=0.30^7^ | *β*=-0.21, *p*=0.38 | *β*=-0.22, *p*=0.40 | *β*=-0.09, *p*=0.69 |
| ASD, autism spectrum disorder; CHR, clinical high risk; PQ-16, Prodromal Questionnaire-16.  ^1^ Covariates: age, sex, smoking-status (yes/no), PET/CT-scanner type (Vereos or Biograph Horizon)  ^2^ *n*=3 (all ASD)  ^3^ *n*=41  ^4^ *n*=24  ^5^ *n*=31  ^6^ *n*=17  ^7^ As reported in the main article. | | | | | |

| **Supplement 4.9. Associations between striatal *k*_i_^cer^ and measures of depressed mood and anxiety.** | | | | | |
| --- | --- | --- | --- | --- | --- |
|  |  | **Striatal region (*k*_i_^cer^)** | | | |
| **Analysis** | **Covariates^1^** | Whole striatum | Associative | Limbic | Sensorimotor |
| BDI-II in ASD | Non-adjusted | *β*=-0.17, *p*=0.27 | *β*=-0.20, *p*=0.19 | *β*=-0.11, *p*=0.46 | *β*=-0.26, *p*=0.09 |
|  | Adjusted | *β*=-0.11, *p*=0.51 | *β*=-0.12, *p*=0.46 | *β*=-0.04, *p*=0.81 | *β*=-0.19, *p*=0.22 |
| BDI-II in controls | Non-adjusted | *β*=-0.02, *p*=0.93 | *β*=-0.05, *p*=0.82 | *β*=0.00, *p*=1.00 | *β*=-0.04, *p*=0.85 |
|  | Adjusted | *β*=0.01, *p*=0.97 | *β*=0.05, *p*=0.83 | *β*=0.00, *p*=0.99 | *β*=0.15, *p*=0.45 |
| STAI-T in ASD | Non-adjusted | *β*=0.12, *p*=0.46 | *β*=0.10, *p*=0.54 | *β*=0.20, *p*=0.18 | *β*=-0.04, *p*=0.80 |
|  | Adjusted | *β*=0.13, *p*=0.40 | *β*=0.12, *p*=0.43 | *β*=0.23, *p*=0.13 | *β*=-0.02, *p*=0.87 |
| STAI-T in controls | Non-adjusted | *β*=-0.14, *p*=0.52 | *β*=-0.10, *p*=0.67 | *β*=-0.20, *p*=0.38 | *β*=0.03, *p*=0.88 |
|  | Adjusted | *β*=-0.20, *p*=0.37 | *β*=-0.15, *p*=0.47 | *β*=-0.24, *p*=0.28 | *β*=-0.02, *p*=0.93 |
| ASD, autism spectrum disorder; CHR, clinical high risk; BDI-II, Beck’s Depression Inventory-II; STAI-T, State-Trait Anxiety Inventory-Trait subscale.  ^1^ Covariates: age, sex, smoking-status (yes/no), PET/CT-scanner type (Vereos or Biograph Horizon) | | | | | |

# **Supplementary References**

1. Kapur S. et al. A positron emission tomography study of quetiapine in schizophrenia: a preliminary finding of an antipsychotic effect with only transiently high dopamine D2 receptor occupancy. *Arch. Gen. Psychiatry* **57**(6), 553-559 (2000).

2. Russell D. W. UCLA Loneliness Scale (Version 3): Reliability, validity, and factor structure. *J. Pers. Assess.* **66**(1), 20-40 (1996).

3. Selten J.-P., van der Ven E., Rutten B. P., Cantor-Graae E. The social defeat hypothesis of schizophrenia: an update. *Schizophr. Bull.* **39**(6), 1180-1186 (2013).

4. Gevonden M. et al. Increased release of dopamine in the striata of young adults with hearing impairment and its relevance for the social defeat hypothesis of schizophrenia. *JAMA Psychiatry* **71**(12), 1364-1372 (2014).

5. Gilman R., Carter-Sowell A., DeWall C. N., Adams R. E., Carboni I. Validation of the ostracism experience scale for adolescents. *Psychol. Assess.* **25**(2), 319 (2013).

6. Olweus D. Revised Olweus bully/victim questionnaire. *Br. J. Educ. Psychol*. (1996).

7. Lubben J. et al. Performance of an abbreviated version of the Lubben Social Network Scale among three European community-dwelling older adult populations. *Gerontologist* **46**(4), 503-513 (2006).

8. Bernstein D. P. et al. Development and validation of a brief screening version of the Childhood Trauma Questionnaire. *Child Abuse Negl*. **27**(2), 169-190 (2003).

9. Thombs B. D., Bernstein D. P., Lobbestael J., Arntz A. A validation study of the Dutch Childhood Trauma Questionnaire-Short Form: factor structure, reliability, and known-groups validity. *Child Abuse Negl.* **33**(8), 518-523 (2009).

10. Spinhoven P. et al. Childhood Trauma Questionnaire: Factor structure, measurement invariance, and validity across emotional disorders. *Psychol. Assess.* **26**(3), 717 (2014).

11. Leary M. R., Kelly K. M., Cottrell C. A., Schreindorfer L. S. Construct validity of the need to belong scale: Mapping the nomological network. *J. Pers. Assess.* **95**(6), 610-624 (2013).

12. Cohen S., Mermelstein R., Kamarck T., Hoberman H. M. In ***Social Support: Theory, Research and Applications*** (eds Sarason I. G., Sarason B. R.) (Martinus Nijhoff Publishers, Dordrecht, 1985).

13. Yung A. R. et al. Mapping the onset of psychosis: the comprehensive assessment of at-risk mental states. *Aust. N. Z. J. Psychiatry* **39**(11-12), 964-971 (2005).

14. Ising H. K. et al. The validity of the 16-item version of the Prodromal Questionnaire (PQ-16) to screen for ultra high risk of developing psychosis in the general help-seeking population. *Schizophr. Bull.* **38**(6), 1288-1296 (2012).

15. Loewy R. L., Bearden C. E., Johnson J. K., Raine A., Cannon T. D. The prodromal questionnaire (PQ): preliminary validation of a self-report screening measure for prodromal and psychotic syndromes. *Schizophr. Res.* **79**(1), 117-125 (2005).

16. Hammers A. et al. Three‐dimensional maximum probability atlas of the human brain, with particular reference to the temporal lobe. *Hum. Brain. Mapp.* **19**(4), 224-247 (2003).

17. Tziortzi A. C. et al. Connectivity-based functional analysis of dopamine release in the striatum using diffusion-weighted MRI and positron emission tomography. *Cereb. Cortex* **24**(5), 1165-1177 (2014).

18. Jenkinson M., Beckmann C. F., Behrens T. E., Woolrich M. W., Smith S. M. Fsl. *Neuroimage* **62**(2), 782-790 (2012).

19. McCarthy P. FSLeyes. https://users.fmrib.ox.ac.uk/~paulmc/fsleyes/userdoc/latest/index.html (2019, August 7).
